# 以出血为主要表现的遗传性蛋白C缺乏症1例并文献复习

**DOI:** 10.3760/cma.j.cn121090-20241129-00494

**Published:** 2024-12

**Authors:** 丹丹 余, 新岳 代, 葳 刘, 玉华 王, 仁池 杨, 磊 张, 峰 薛

**Affiliations:** 1 中国医学科学院血液病医院（中国医学科学院血液学研究所），血液与健康全国重点实验室，国家血液系统疾病临床医学研究中心，细胞生态海河实验室，天津市血液病基因治疗研究重点实验室，中国医学科学院血液病基因治疗重点实验室，天津 300020 State Key Laboratory of Experimental Hematology, National Clinical Research Center for Blood Diseases, Haihe Laboratory of Cell Ecosystem, Tianjin Key Laboratory of Gene Therapy for Blood Diseases, CAMS Key Laboratory of Gene Therapy for Blood Diseases, Institute of Hematology & Blood Diseases Hospital, Chinese Academy of Medical Sciences & Peking Union Medical College, Tianjin 300020, China; 2 天津医学健康研究院，天津 301600 Tianjin Institutes of Health Science, Tianjin 301600, China; 3 中国医学科学院北京协和医学院群医学及公共卫生学院，北京 100730 Peking Union Medical College, Beijing 100730, China

**Keywords:** 遗传性蛋白C缺乏症, 弥散性血管内凝血, 抗凝, Hereditary protein C deficiency, Disseminated intravascular coagulation, Anticoagulation

## Abstract

**目的:**

加深对遗传性蛋白C缺乏症的了解。

**方法:**

报告1例以反复肌肉出血为主要临床表现的重型遗传性蛋白C缺乏患儿的诊疗过程，并进行相关文献复习。

**结果:**

患儿，女，2岁，因“反复肌肉血肿2年余、颅内出血3个月”入院。查体见左侧小腿可见皮肤淤青，触诊局部皮温升高。双下肢肌力及肌张力无明显异常，左侧大腿围35.6 cm，右侧大腿围29 cm。实验室检查示纤维蛋白原、凝血因子XIII活性及蛋白C活性降低，纤维蛋白降解产物（FDP）和D-二聚体水平明显增高。基因检测提示PROC基因复合杂合突变c.565（exon7）C>T和c.983_988（exon7）del GCGAGC。诊断为遗传性蛋白C缺乏症、弥散性血管内凝血，予补充纤维蛋白原、新鲜冰冻血浆及联合抗凝治疗，病情好转。

**结论:**

遗传性蛋白C缺乏患儿临床表现异质性大，除了引起常见的新生儿暴发性紫癜外，也可以反复出血为主要临床表现。

## Abstract (en)

**Objective:**

To enhance the understanding of hereditary protein C deficiency.

**Methods:**

A case of a child with severe hereditary protein C deficiency, presenting with recurrent muscle bleeding as the primary clinical manifestation, was reported. The diagnostic and treatment were detailed, and relevant literature was reviewed.

**Results:**

The patient, a 2-year-old girl, was admitted with “recurrent muscle hematomas for more than 2 years and intracranial hemorrhage for 3 months.” Ecchymosis was observed on the skin of the left calf, with localized warmth detected on palpation. Muscle strength and tone in both lower limbs were within normal limits. The left thigh circumference measured 35.6 cm, compared to 29 cm on the right. Laboratory tests showed decreased fibrinogen, coagulation factor XIII activity, protein C activity, and significantly elevated fibrin degradation products（FDP）and D-dimer levels. Genetic testing identified compound heterozygous mutations in the PROC gene: c.565（exon 7）C>T and c.983_988（exon 7）del GCGAGC. The patient was diagnosed with hereditary protein C deficiency and disseminated intravascular coagulation（DIC）. Treatment with fibrinogen supplementation, fresh frozen plasma, and anticoagulation therapy led to clinical improvement.

**Conclusion:**

The clinical manifestations of hereditary protein C deficiency are highly heterogeneous. While neonatal purpura fulminans is a common presentation, recurrent bleeding can also serve as a primary clinical manifestation.

蛋白C（Protein C, PC）是一种维生素K依赖性丝氨酸蛋白酶原，主要在肝脏中合成。在内皮细胞表面，PC通过凝血酶—血栓调节蛋白复合物的作用被激活为活化蛋白C（APC）。APC进一步与辅因子蛋白S结合形成复合物，通过灭活凝血因子Ⅴa和Ⅷa，抑制凝血酶的生成，从而发挥抗凝功能[1]。1981年，Griffin等[2]首次报道了遗传性蛋白C缺乏症，并指出其与静脉血栓形成密切相关。遗传性蛋白C缺乏症是一种由PROC基因突变引起的常染色体显性遗传病，可导致蛋白C数量减少（Ⅰ型）或功能异常（Ⅱ型），是中国人群中最常见的遗传性易栓症类型[3]–[5]。PROC基因单一杂合突变较为常见，发病率1/200～1/500，患者通常表现为血栓形成风险增高，约5％的静脉血栓形成（VTE）患者携带PROC基因杂合突变[6]。复合杂合突变或纯合突变导致的严重遗传性蛋白C缺乏症极为罕见，发病率为1/50万～1/75万，多在新生儿时期引发暴发性紫癜及弥散性血管内凝血（DIC），病情进展迅速且病死率高[7]。本文报道1例以反复肌肉出血为主要临床表现的遗传性复合杂合突变引起的重型蛋白C缺乏患儿，并从发病机制、临床表现、诊断及治疗等方面对该病进行文献复习，旨在提高临床医生对该病的认识，作到早期诊断和合理治疗。

## 病例资料

患儿，女，2岁，2022年12月因“反复肌肉血肿2年余、颅内出血3个月”入院。患儿自4月龄起反复出现下肢瘀斑伴肿胀，初诊时在当地医院检查发现纤维蛋白原（FIB）显著降低（0.59 g/L），给予间断纤维蛋白原输注治疗，但仍有间歇性出血。2022年9月，患儿因外伤后硬膜外血肿拟行手术治疗，术前凝血功能检查显示纤维蛋白原0.35 g/L。输注纤维蛋白原后实施手术，术后输注纤维蛋白原1 g/d。术后第13天，患儿右侧大腿皮肤出现坏死。诊断为弥散性血管内凝血（DIC）及脓毒性休克，经过局部换药、抗感染、抗休克、肝素抗凝及冷沉淀治疗后病情有所好转。患儿出院后仍出现反复下肢瘀斑及肿胀，复查凝血功能提示：活化部分凝血活酶时间（APTT）58.7 s，凝血酶原时间（PT）46.6 s，凝血酶时间（TT）23.7 s，为进一步治疗就诊于我院。患儿系第2胎第2产，其母亲第1胎于出生后42 d因“化脓性脑膜炎、高胆红素血症、凝血功能障碍”去世。家族其他成员体健，否认家族相关病史及遗传病史。

入院体格检查：体温36.8 °C，身高95 cm，体重12 kg，脉率115次/分，呼吸26次/分，血压96/69 mmHg。神志清，发育正常，营养中等，一般状态良好；颅脑枕部可见长约5 cm皮肤瘢痕；右下肢及臀部多发皮肤溃疡后结痂、瘢痕，左侧小腿可见皮肤淤青，局部皮温升高。双下肢肌力及肌张力无明显异常，左大腿围35.6 cm，右大腿围29 cm。

入院血常规：WBC 5.72×10^9^/L，中性粒细胞58.7％，HGB 101 g/L，PLT 271×10^9^/L；凝血功能：APTT 39.2 s（参考值22.6～32.1 s），PT 18.2 s（参考值10.0～14.0 s），TT 34.7 s（参考值13.3～19.3 s），FIB 0.25 g/L（参考值2.00～4.00 g/L），FDP 224.7 mg/L（参考值<5.0 mg/L），D-二聚体>80.00 mg/L（参考值0～0.55 mg/L）；血栓两项：凝血酶-抗凝血酶复合物（TAT）>120.00 µg/L（参考值<4.08 µg/L），纤溶酶-α2纤溶酶抑制剂复合物（PIC）12.31 mg/L（参考值<0.85 mg/L）；凝血因子Ⅷ活性（FⅧ∶C）25.3％（参考值50.0％～150.0％），凝血因子XIII活性（FXIII∶C）4.3％（参考值50.0％～120.0％）；纤溶酶原103.1％（参考值75.0％～150.0％），α2纤溶酶抑制物89％（参考值80％～120％）；蛋白C 1.1％（参考值70.0％～140.0％），蛋白S 81.4％（参考值70.0％～123.0％）；肝功能、肾功能、电解质等正常。双下肢血管超声未见确切血栓形成。

入院后初步诊断为蛋白C缺乏合并DIC，立即予纤维蛋白原0.5 g输注，同时予普通肝素35 IU/kg每6 h 1次静脉注射。次日，予以补充新鲜冰冻血浆（FFP）10 mg/kg每12 h 1次输注，连用2 d。患儿皮肤瘀斑及肿胀较前减轻，3 d后复查FIB明显上升，D-二聚体显著下降。后将抗凝方案改为低分子肝素0.1 ml/10 kg（体重）每12 h 1次皮下注射，疗效欠佳。随后改为口服利伐沙班抗凝治疗，经剂量调整（5 mg每日1次→5 mg每日2次→3.33 mg每日3次），患儿病情得到有效控制。住院治疗期间全外显子基因检测：PROC基因存在复合杂合突变，包括7号外显子c.565C>T（p.Arg189Trp）和9号外显子c.983_988delGCGAGC（p.Arg328_Glu329del），两处突变分别源于母亲（exon7 c.565C>T, p.Arg189Trp）和父亲（exon9 c.983_988delGCGAGC, p.Arg328_Glu329del）。明确诊断为遗传性蛋白C缺乏症。出院后继续口服利伐沙班抗凝治疗（3.33 mg每日3次）。随访至今，患儿仍有间断皮肤瘀斑表现，间断予以FFP输注。

## 讨论及文献复习

编码蛋白C的PROC基因位于染色体2q13-q14，全长11.2 kb，由9个外显子和8个内含子组成[8]。截止目前，人类基因突变数据库（HGMD）已报道超过500种PROC基因突变，不同类型及位点的突变引起血栓形成的风险不同[9]。本例患儿携带来源于母亲的7号外显子突变位点c.565C>T（p.Arg189Trp），这是我国常见的血栓形成危险因素。该突变可导致蛋白C结构改变，进而阻碍其与蛋白S、磷脂等的相互作用，导致蛋白C功能异常，并使血栓栓塞的发生风险增加5～7倍[10]。此外，患儿还携带源于父亲的9号外显子突变c.983_988delGCGAGC（p.Arg328_Glu329del），该突变类型尚未见相关文献报道。患儿父母双方均无相关临床表现，可能与疾病的外显率相关。该患儿为上述两个位点的复合型突变携带者，突变的协同作用显著提高了患病风险，最终导致疾病发生。

由复合杂合突变引起的重型遗传性蛋白C缺乏患儿可在出生后6～24 h即出现症状，典型表现为以微血管栓塞为特征的暴发性紫癜、DIC及多发脏器血栓栓塞。该病病情进展迅速，病死率高。目前国内报道的重型遗传性蛋白C缺乏病例多以新生儿暴发性紫癜起病[11]–[13]。

本例患儿于4月龄起病，以反复肌肉出血为主要临床表现，显著不同于重型遗传性蛋白C缺乏症患儿常见的血栓倾向，增加了诊断难度并导致诊断延迟。在完成蛋白C水平测定及PROC基因突变分析后，方获得明确诊断。该患儿由于体内蛋白C重度缺乏，导致凝血系统持续激活，凝血因子消耗性降低，进而引起持续性慢性纤溶亢进，纤维蛋白原明显降低，表现出类似于DIC的临床表征，临床表现以严重出血为主。此外，患儿明确诊断时也伴有FXIII消耗性降低，虽然没有首次发病时数据，但是由于该病是一个慢性过程，推测在病程中会持续存在获得性FXIII缺乏，与纤维蛋白原降低共同导致出血。而在持续输注大量纤维蛋白原后，凝血系统进一步激活，患儿反而出现暴发性紫癜。这一现象符合遗传性蛋白C缺乏症的病理生理机制，即在凝血和纤溶动态平衡紊乱的背景下，血栓形成风险与出血表现可相继出现。这也提示临床医生应充分认识该病的临床表现异质性，同时重视蛋白C水平检测及基因检测等检查在该病诊断中的关键作用，以早期诊断和积极治疗。

遗传性蛋白C缺乏患儿的最佳治疗方案为蛋白C终生替代治疗，但目前国内尚无法获得蛋白C浓缩物，目前主要依赖长期抗凝治疗进行维持，同时按需补充FFP和相关凝血因子以控制急性症状。对于急性期患儿，应尽早实施干预治疗，推荐使用FFP 10～15 ml/kg每8～12 h输注1次，直至临床症状缓解、病变消退及D-二聚体等实验室指标恢复正常。在此基础上，可联合抗凝治疗进一步控制凝血系统的异常激活并预防后续血栓事件的发生[14]。

低分子肝素（LMWH）因其药代动力学特性更加可预测，已成为儿科血栓治疗的一线药物[15]。然而，本例患儿应用LMWH效果不佳。华法林是遗传性血栓性疾病二级预防的基础用药，1988年首次报道华法林治疗8月龄遗传性蛋白C患儿，并提出华法林长期治疗纯合突变遗传性蛋白C缺乏症的有效性[16]。然而，需警惕华法林所致皮肤坏死（WISN）这一并发症，常发生于治疗初期，尤其在重型遗传性蛋白C缺乏患儿中更为常见[17]。Menon等[18]曾报道口服利伐沙班成功治疗一例13岁重型遗传性蛋白C缺乏患儿的WISN。近年来，利伐沙班作为口服直接凝血因子Xa抑制剂，且不需要频繁监测凝血功能，逐渐成为儿童抗凝治疗的新选择。一项多中心随机对照试验（RCT）表明，与传统标准治疗相比，利伐沙班治疗儿童VTE后复发率更低（1.2％对3.0％）且未发生严重出血事件[19]。遗传性蛋白C缺乏作为一种罕见病，患儿间存在显著个体差异，抗凝治疗方案需根据患儿具体情况进行个性化调整。对于体重≥12 kg的儿童，利伐沙班治疗VTE的常规推荐剂量为5 mg每日2次[20]。考虑本例患儿同时存在出血风险，初始采用低于常规剂量的利伐沙班（5 mg每日1次），后根据病情及实验室指标动态调整剂量，最终病情得以有效控制。

肝移植目前被认为是重型遗传性蛋白C缺乏患儿的唯一治愈手段。国外已有多个肝移植治疗该病的病例报道，患儿通常具有良好的长期生存率[21]–[24]，国内尚无相关病例报道。然而，肝移植作为一种侵入性治疗措施，需全面权衡其潜在的长期风险与患儿的预期获益。

## References

[1] Dinarvand P, Moser KA (2019). Protein C Deficiency[J]. Arch Pathol Lab Med.

[2] Griffin JH, Evatt B, Zimmerman TS (1981). Deficiency of protein C in congenital thrombotic disease[J]. J Clin Invest.

[3] Cooper PC, Hill M, Maclean RM (2012). The phenotypic and genetic assessment of protein C deficiency[J]. Int J Lab Hematol.

[4] Tang L, Wang HF, Lu X (2013). Common genetic risk factors for venous thrombosis in the Chinese population[J]. Am J Hum Genet.

[5] 全 睿琳, 何 建国 (2019). 亚洲人群静脉血栓栓塞症相关基因多态性研究进展[J]. 中华医学杂志.

[6] Bruley DF (2007). Anticoagulant blood factor deficiencies (protein C)[J]. Adv Exp Med Biol.

[7] Goldenberg NA, Manco-Johnson MJ (2008). Protein C deficiency[J]. Haemophilia.

[8] Khor B, Van Cott EM (2010). Laboratory tests for protein C deficiency[J]. Am J Hematol.

[9] Zhang N, Sun DK, Tian X (2024). Protein C deficiency with venous and arterial thromboembolic events[J]. World J Clin Cases.

[10] Ding Q, Shen W, Ye X (2013). Clinical and genetic features of protein C deficiency in 23 unrelated Chinese patients[J]. Blood Cells Mol Dis.

[11] 汤 普森, 张 志波 (2021). 蛋白C缺乏症导致的新生儿暴发性紫癜一例并文献复习[J]. 中国小儿急救医学.

[12] 段 袁园, 吴 优, 许 愿愿 (2022). 遗传性蛋白C缺陷症致新生儿暴发性紫癜2例报告及文献复习[J]. 中华血液学杂志.

[13] 宋 予晴, 肖 娟, 唐 晓艳 (2022). 口服抗凝剂治疗以新生儿暴发性紫癜或颅内出血为首发表现的遗传性复合杂合突变的重度蛋白C缺乏症2例病例报告并文献复习[J]. 中国循证儿科杂志.

[14] Minford A, Brandão LR, Othman M (2022). Diagnosis and management of severe congenital protein C deficiency (SCPCD): Communication from the SSC of the ISTH[J]. J Thromb Haemost.

[15] Law C, Raffini L (2015). A guide to the use of anticoagulant drugs in children[J]. Paediatr Drugs.

[16] Peters C, Casella JF, Marlar RA (1988). Homozygous Protein C Deficiency: Observations on the Nature of the Molecular Abnormality and the Effectiveness of Warfarin Therapy[J]. Pediatrics.

[17] Chan YC, Valenti D, Mansfield AO (2000). Warfarin induced skin necrosis[J]. Br J Surg.

[18] Menon N, Sarode R, Zia A (2018). Rivaroxaban dose adjustment using thrombin generation in severe congenital protein C deficiency and warfarin-induced skin necrosis[J]. Blood Adv.

[19] Male C, Lensing AWA, Palumbo JS (2020). Rivaroxaban compared with standard anticoagulants for the treatment of acute venous thromboembolism in children: a randomised, controlled, phase 3 trial[J]. Lancet Haematol.

[20] Jaffray J, Young G (2022). Direct oral anticoagulants for use in paediatrics[J]. Lancet Child Adolesc Health.

[21] Lee MJ, Kim KM, Kim JS (2009). Long-term survival of a child with homozygous protein C deficiency successfully treated with living donor liver transplantation[J]. Pediatric Transplantation.

[22] Angelis M, Pegelow CH, Khan FA (2001). En bloc heterotopic auxiliary liver and bilateral renal transplant in a patient with homozygous protein C deficiency[J]. J Pediatr.

[23] Boucher AA, Luchtman-Jones L, Nathan JD (2018). Successful liver transplantation for homozygous protein C deficiency with a type II mutation using a heterozygous living related donor[J]. Am J Hematol.

[24] Monagle K, Ignjatovic V, Hardikar W (2014). Long-term follow-up of homozygote protein C deficiency after multimodal therapy[J]. J Pediatr Hematol Oncol.

